# STC2 promotes the epithelial-mesenchymal transition of colorectal cancer cells through AKT-ERK signaling pathways

**DOI:** 10.18632/oncotarget.12147

**Published:** 2016-09-20

**Authors:** Bing Chen, Xiao Zeng, Yu He, Xixi Wang, Ziwei Liang, Jingjing Liu, Peng Zhang, Hongxia Zhu, Ningzhi Xu, Shufang Liang

**Affiliations:** ^1^ State Key Laboratory of Biotherapy and Cancer Center, West China Hospital, Sichuan University, and Collaborative Innovation Center for Biotherapy, Chengdu, 610041, P.R. China; ^2^ Department of Urinary Surgery, West China Hospital, Sichuan University, Chengdu, 610041, Sichuan, P. R. China; ^3^ Laboratory of Cell and Molecular Biology & State Key Laboratory of Molecular Oncology, Cancer Institute & Cancer Hospital, Chinese Academy of Medical Sciences, Beijing, 100034, P. R. China

**Keywords:** stanniocalcin 2, colorectal cancer, epithelial-mesenchymal transition, tumor biomarker

## Abstract

The STC2 protein involves in carcinogenesis and progression of many cancers. It remains unclear how STC2 regulates the epithelial-mesenchymal transition (EMT) process and colorectal cancer (CRC) development. Here we systematically investigated STC2-activated early occurrence of EMT and CRC cell migration *in vitro*, clinical associations of STC2 with CRC development and patient survival. The secretion and expression level of STC2 were both greatly increased in EMT cells and CRC cells compared with the normal epithelial NCM460 cells. And the conditioned media from EMT cells stimulated epithelia and colon cancer cells to obtain EMT characteristics. STC2 overexpression promoted CRC cell growth and cell migration *in vitro*, and STC2 enhanced tumor growth in a mouse CRC-xenograft model. Corresponding to EMT marker expression changes, several critical signaling pathway molecules including pERK, pAKT, PI3K and Ras were remarkably increased either in NCM460 cells transfected with STC2 plasmids or in cells induced with exogenous STC2 protein. However blocking AKT-ERK signaling pathways attenuated STC2-activated EMT process. Furthermore the elevated STC2 expressions were also confirmed in 77 clinical tumor tissues and sera from CRC patients, and the increased STC2 in tumor tissues and sera correlated with tumor pathologic stage and poor survival for CRC patients. In conclusion, STC2 promotes CRC tumorigenesis and EMT progression through activating ERK/MEK and PI3K/AKT signaling pathways. STC2 protein is also a potential tumor biomarker for CRC diagnosis and prognosis.

## INTRODUCTION

The stanniocalcin (STC) family consists of stanniocalcin 1 (STC1) and stanniocalcin 2 (STC2), which are glycoproteins as hormones to regulate calcium and phosphate secretion [1]. STC was previously reported to be present in the corpuscle of stannius, an endocrine gland of bony fish associated with Ca^2+^ homeostasis [2]. Lately, STC1 and STC2 have been found to widely express in various human tissues [3]. Except as a calcium and phosphate regulator, STC2 exhibits potent growth-suppressive properties and participates in bone development [4]. STC2 up-regulation can be caused by the unfolded-protein response in eukaryotic cells through activating transcription factor 4 after activation of the endoplasmic reticulum kinase [5, 6]. Its roles in pancreatic cell injury were further discovered [7].

More importantly, STC2 has been reported to involve in progression of many cancers. For example, a higher expression level of STC2 was detected in gastric cancer [8]. Significantly an up-regulated expression of STC2, which is a target of retinoic acid signaling pathway in breast cancer, has been identified [9]. Ieta had detected high STC2 mRNA expression in colorectal cancer [10]. Consistently, the up-regulation of STC2 both at mRNA and protein level is linked with a shorter survival rate in renal cell carcinoma [11]. And STC2 location is also different between normal renal tissues and renal tumors. In renal cancer this protein is strongly expressed in cytoplasm and cell membrane. These investigations suggest that STC2 has a close relationship with several types of cancer.

Increasing evidences indicate the epithelial-mesenchymal transition (EMT), a conserved process linked with embryonic development, also plays essential roles in carcinogenesis and tumor progression [12, 13]. The first and determinant step of the EMT process is a local invasion through the epithelial basement membrane, because it requires modifications in cell-cell and cell-matrix interactions, as well as enhancement of cell motility [13, 14]. The essence of the EMT process is that epithelia acquired mesenchymal features including decreasing cell adhesion and increasing cell motility, which is a main step of tumor metastasis. Within tumor microenvironment, both tumors and stromal cells could secrete a series of proteins to regulate EMT process [14]. In our previous study, we found the expression of STC2 secreted by colon mucosal epithelial cells was dynamically altered within tumor microenvironment *via* a SILAC-based quantitative proteomic analysis [15], which indicates STC2 has important roles in cell-cell interactions between tumor cells and normal colon epithelia. In addition, colorectal cancer (CRC) has been a model of tumor progression for several years, and EMT is a dominant program in human colon cancer [16]. Therefore, the biological functions of STC2 in EMT process and colon cancer development deserve to further explore in detail.

In the present study, we systematically investigated STC2-mediated early occurrence of EMT and its involvement of colon cancer migration, clinical associations of STC2 level with tumor development stages and CRC patient survival, as well as discovered STC2 functions on CRC tumorigenesis and progression by promoting EMT process through activating ERK/MEK and PI3K/AKT signaling pathways.

## RESULTS

### Colon epithelial cells are induced into EMT- featured cells

In order to obtain colon cells with EMT features, also named as EMT cells, human colon mucosal epithelial NCM460 cells were induced into EMT cells by continuously treated with phorbol-12-myristate-13-acetate (PMA), which had been used as an EMT inducer for human prostate cancer cells [17].

**Figure 1:**
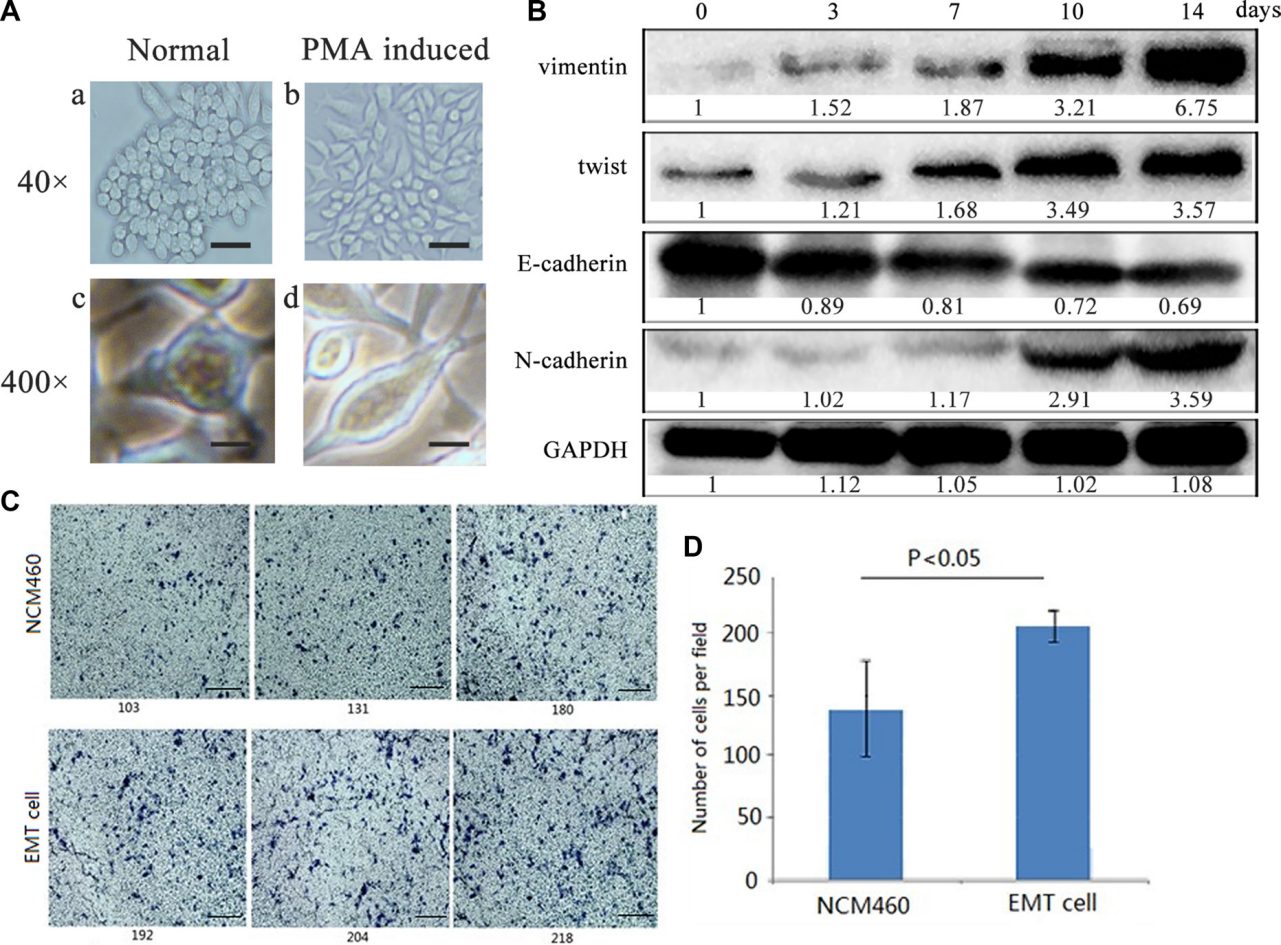
Colon epithelial NCM460 cells were induced into EMT- featured cells by PMA treatment (**A**) Cell morphology changes of NCM460 cells induced by PMA. NCM460 cells displayed epithelial morphology (a, c), while those NCM460 cells, which were treated with 100 ng/ml PMA for 24 h, showed spindle-like mesenchymal morphology (b, d). The scale bar respectively represents 100 μm (a, b) at 40×magnification, and 10 μm (c, d) at 400×magnification of phase contrast microphotographs. (**B**) EMT biomarkers were dynamically expressed in PMA-induced NCM460 cells at different treatment days. EMT cells were obtained from PMA-induced NCM460 cell clones after selection for 8 cell passages (14 days). (**C**) Cell invasion ability was greatly increased in EMT cells than normal NCM460 cells (*P* < 0.05). Cells were grown on the matrigel for 72 h. The scale bar represents 300 μm, with 400×magnification. (**D**) Data are representative of at least three independent experiments. The average values ± the standard error of the mean (SEM) of three experiments were plotted.

We originally used 10–1000 ng/ml PMA to treat NCM460 cells for 24 hours to determine an optimal concentration. And 100 ng/ml PMA in the medium could alter cell growth of NCM460 and also sustain cell vitality. Under the conditions, NCM460 cells were stepwise changed from cobblestone-like to spindle-like shapes and became dissociated from each other after PMA treatment for 24 hours (Figure 1A–1B, 1D). To determine the time-dependent changes of the EMT markers in PMA-induced NCM460 cells, we detected N-cadherin, E-cadherin, vimentin and twist in NCM460 cells with 100 ng/ml PMA exposure respectively for 0, 3, 7, 10, and 14 days. As expected several key EMT markers showed time-dependent changes. The N-cadherin, vimentin and twist were all gradually up-regulated in NCM460 cells treated with 100 ng/ml PMA, while the E-cadherin level was stepwise decreased (Figure 1B). Indeed, the time of 5, 7 cell passages was almost same as PMA treatment for 10, 14 days respectively. Therefore the EMT cells were acquired from stable cell clones with EMT features, including morphology of mesenchymal stromal cells and EMT biomarker expression, after a continuous PMA stimulation for 8 cell passages. Besides cell-cell dissociation, loss of cellular polarity and spindle-like shapes, cell invasion ability of EMT cells (Figure 1C–1D) was significantly higher than NCM460 cells (*P* < 0.05). All above results indicate that the colon epithelia-derived EMT cells were established successfully.

### Conditioned media from EMT cells stimulate epithelia and colon cancer cells to obtain EMT characterization

**Figure 2:**
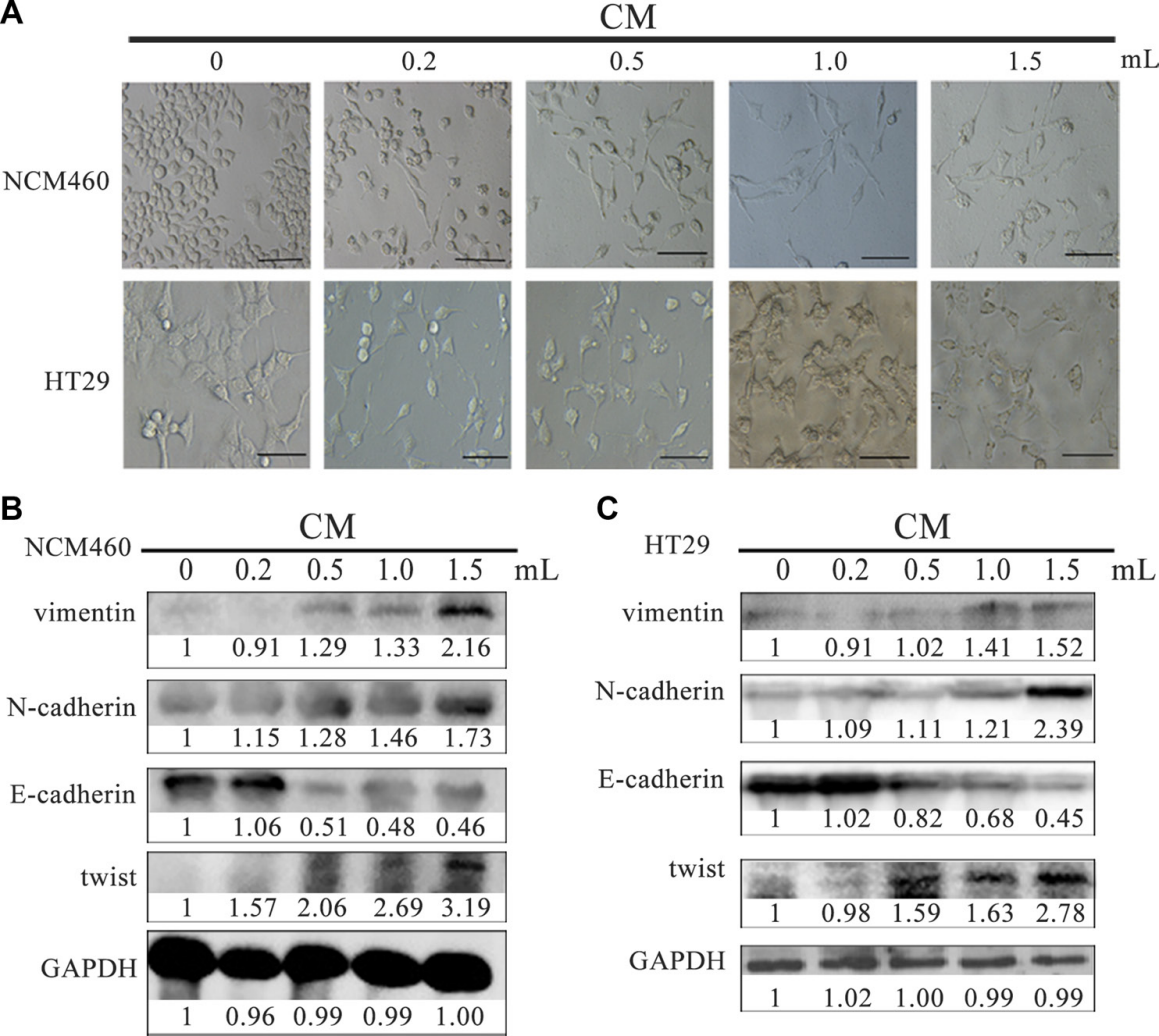
CM derived from EMT cells induced NCM460 and HT29 cells to exhibit EMT features Cell phenotype (**A**) and EMT biomarker expression (**B**, **C**) of NCM460 and HT29 cells incubated with EMT cell-derived CM for 24 h. The scale bar represents 10 μm, with 400×magnification. CM: conditioned media.

In order to investigate biological influences of total proteins secreted by EMT cells, firstly we collected the conditioned media (CM) supernatants from EMT cells to treat normal colon epithelial NCM460 cells to compare cell phenotypes and molecular expression changes. When 0.2 ml of 0.8 μg/μl CM was added to incubate with NCM460 cells for 24 hours, the treated NCM460 cells exhibited spindle-like shapes, and cell connection was no longer tightly. With a higher amount (from 0.5, 1.0 to 1.5 ml) of 0.8 μg/μl CM to incubate, NCM460 cells were gradually induced into EMT phenotypes from epithelial to mesenchymal shapes, and cells became scattering distributed (Figure 2A up). Moreover different quantity (from 0.2 to1.5 ml) of 0.8 μg/μl CM was incubated with colon cancer HT29 cells for 24 hours, and similar morphology changes were observed too (Figure 2A down).

Except for morphology investigation, we also detected several EMT markers in NCM460 cells after incubation with CM. The expression of vimentin, N-cadherin and twist were highly increased in NCM460 cells incubated with 1 ml of 0.8 μg/μl CM, while E-cadherin was significantly decreased (Figure 2B).

Similarly, with a higher concentration of CM to add to HT29 cells, the expression of vimentin, N-cadherin and twist was all gradually increased compared with the parent untreated HT29 cells, while E-cadherin was obviously downregulated (Figure 2C). Based on the above results, it indicated CM derived from EMT cells could stimulate epithelia NCM460 and cancer HT29 cells to acquire EMT features, in which some proteins secreted by EMT cells are involved in this biological process. Similar results had been reported in MCF-7 cells which were incubated with CM derived from EMT-featured MCF-7 cells [18].

In addition, to avoid the influence of the different amount (0.2, 0.5, 1.0 to 1.5 ml) of EMT cell-derived CM on the components of the cell culture media for cell growth, we supplied additional volume of fetal bovine serum (FBS) to ensure the final concentration with 10% FBS in 2 mL culture media to observe cell changes. Actually, a little volume of additional FBS almost had no influence on cell growth and EMT molecule expression (Supplementary Figure S1).

### Intracellular expression and secretion of STC2 are increased in EMT and CRC cells

**Figure 3:**
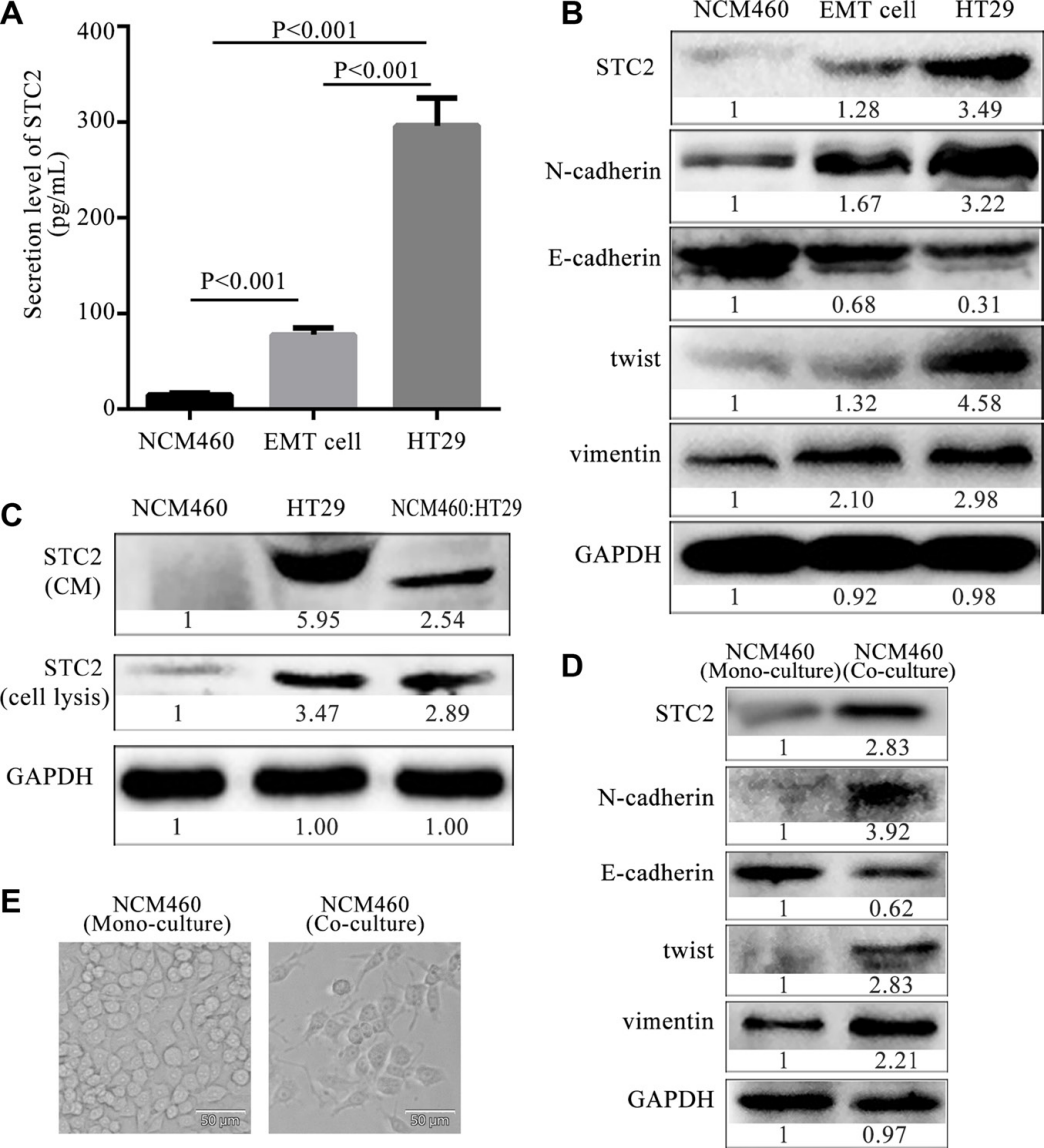
Expression and secretion level of STC2 from epithelia, EMT and colon cancer cells (**A**) The extracellular secretion levels of STC2 from colorectal epithelial NCM460, EMT and HT29 cells. The average values ± SEM was plotted from three separate experiments. (**B**) The intracellular expression of STC2 and EMT markers in NCM460, EMT and HT29 cells. (**C**) Protein secretion and expression of STC2 in NCM460, HT29 and co-cultured NCM460: HT29 cells. (**D**) The expression of STC2 and EMT markers in co- cultured NCM460 cells compared with the mono-cultured NCM460 cells. (**E**) Cell morphology of co-cultured NCM460 cells compared with the mono-cultured NCM460 cells (400×, the scale bar represents 50 μm).

We investigated if there were different intracellular expression and secretion levels of STC2 between normal epithelial cells and EMT cells. Firstly, the secretion levels of STC2 in the culture of NCM460, EMT and HT29 cells were measured by ELISA. The concentration of STC2 secreted by EMT cells was almost increased by 4 folds compared with NCM460 cells (76.53 ± 9.75 pg/ml versus 14.87 ± 3.57 pg/ml, *P* < 0.001), and the secretion level of STC2 by HT29 cells was approximately increased by 19 folds than NCM460 cells (296.45 ± 28.64 pg/ml versus 14.87 ± 3.57 pg/ml, *P* < 0.001) (Figure 3A). The data indicated that CRC and EMT cells secrete more STC2 proteins than same amounts of normal colon epithelial cells. At the same time, the intracellular expression of STC2 was gradually increased from NCM460, EMT and HT29 cells (Figure 3B). Moreover, corresponding to a much higher level of STC2 protein, the EMT markers (N-cadherin, twist and vimentin) were also obviously higher in HT29 cells than NCM460 and EMT cells, while E-cadherin was much downregulated (Figure 3B). The results implied an up- regulated level of STC2 appeared in cells from epithelial to mesenchymal transition.

In order to further investigate STC2 secretion changes in cell-cell interactions between normal epithelia and colon cancer cells, the intracellular expression and extracellular secretion of STC2 respectively was detected in a co-culture system of NCM460 and HT29 cells (NCM460:HT29) at 1:1 cell number ratio. The co-cultured NCM460:HT29 cells respectively showed 2.54, 2.89-fold increase in STC2 secretion and intracellular expression in comparison to NCM460 cell cultures alone (Figure 3C). Actually, the increase of STC2 secretion in co-cultured NCM460:HT29 cells was mainly released from HT29 cells, and a little of STC2 was secreted from the co-cultured NCM460 cells, which was tracked from the mass spectrometry (MS) analysis (Supplementary Figure S2) in our reported study [15]. The MS quantification for 2.29-folds of STC2 secretion was similar with the 2.54 folds detected by Western botting in co-cultured NCM460:HT29 cells. According to STC2 secretion analyzed by MS, the 2.29-folds of STC2 increase in the co-cultured CM was composed of 0.01 folds of STC2 (SILAC ratio 1), secreted from NCM460 cells (Supplementary Figure S2A), and 2.28 folds of STC2 (SILAC ratio 2) that derived from HT29 cells (Supplementary Figure S2B). The SILAC ratio 1 of STC2 was 0.01, which reflected the relative STC2 secretion from NCM460 cells versus the co-cultured CM (NCM460/co-culture) (Supplementary Figure S2A). While the SILAC ratio 2 of STC2 secretion was 2.28, which derived from HT29 cells versus the co-cultured CM (HT29/co-culture) (Supplementary Figure S2B). Therefore the STC2 level secreted from the co-cultured NCM460:HT29 cells was greatly increased mainly from HT29 cell secretion when compared with the mono-cultured NCM460 cells.

Within tumor microenvironment, tumor-associated epithelia (TAE) are defined as a group of epithelia whose function is pirated by tumor cells and redirected toward carcinogenesis [19]. TAE are different from normal epithelia due to the influence of tumor cells. In the co-cultured NCM460:HT29 cell system, the co-cultured NCM460 cells, which were referred to TAE, underwent EMT changes (Figure 3D–3E) compared with mono-cultured NCM460 cells. Besides an elevated STC2 expression, vimentin, N-cadherin and twist were increased in the co-cultured NCM460 cells, while E-cadherin was obviously decreased (Figure 3D). Moreover, cell morphology also showed spindle-like mesenchymal phenotypes (Figure 3E). Therefore the intracellular expression and secretion level of STC2 are increased in the occurrence process from normal epithelial cells to TAE which was gradually obtained EMT-like phenotypes, even induced into cancer cells.

### STC2 promotes colorectal cancer tumorigenesis *in vitro* and *in vivo*

**Figure 4:**
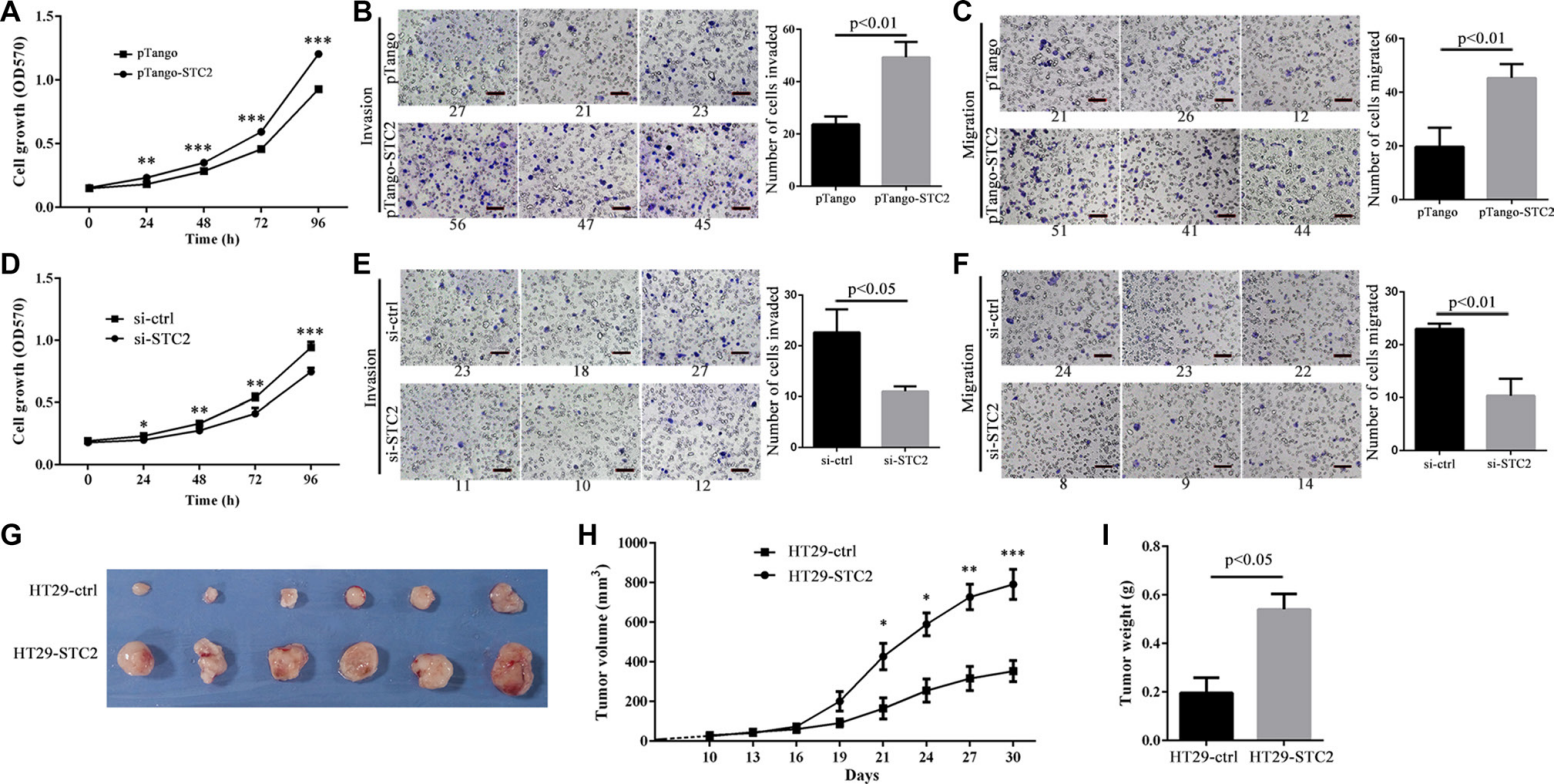
STC2 promotes colorectal cancer tumorigenesis *in vitro* and *in vivo* Cell proliferation, invasion and migration were greatly increased or decreased in STC2-overexpressing (**A–C**) or STC2-knockdown (**D–F**) HT29 cells. Cell migration and cell invasion was observed at 24, 48 h after transfection with pTango-STC2 plasmid (A-C) or STC2 siRNA (si-STC2) (D-F). The average values ± SEM was obtained from three separate experiments. **P < 0.01, ***P < 0.001. (**G**) The tumors isolated from xenograft nude mice (*n* = 6), which were injected with STC2-overexpressing HT29 cells, were much bigger than the control group injected with pTango-containing HT29 cells. After the tumor growth for 20 days (cell injection for 30 days), nude mice were killed to isolate tumor. (**H**) Tumor growth curves of xenograft nude mice. HT29-STC2:STC2-overexpressing HT29 cells. HT29-ctrl: pTango-containing HT29 cells, the control group. The average values ± SEM were calculated from three separate experiments. *P < 0.05, **P < 0.01, ***P < 0.001. (**I**) The average weight of the dissected tumors was much heavier than the control group (*n* = 6).

We investigated the gain- or loss-of-functions of STC2 expression in CRC cells and mouse CRC xenograft models. The STC2-containing pTango-STC2 plasmids were transiently transfected into HT29 cells for 24–96 h, which improved cell proliferation (Figure 4A), cell invasion and migration (Figure 4B–4C). Conversely, STC2 knockdown by STC2-specific siRNA for 48–96 h (Supplementary Figure S3) greatly inhibited cell growth, cell invasion and cell migration of HT29 cells (Figure 4D–4F).

STC2 was further confirmed to enhance tumor growth on CRC-xenograft nude mice. About 10 days after injection, palpable tumors arose approximately with 30 mm^3^, and tumor size was totally monitored for 8 times from then on. The tumor volumes from mice injected with pTango-STC2-containing HT29 cells became larger than the control group after cell injection for 19 days (201.10 ± 84.86 mm^3^ *versus* 91.42 ± 35.30 mm^3^, *P* = 0.108). After cell injection for 21 days, the average tumor size of the STC2-overexpressing mice, with 427.27 ± 115.51 mm^3^, was significantly larger than the control group (164.71 ± 92.98 mm^3^, *P* < 0.05). At the 30 days' injection time, the average tumor size of STC2-overexpressing mice was up to 791.24 ± 267.12 mm^3^, which was more than 3-fold larger than the control (212.38 ± 93.22 mm^3^) (*P* < 0.001, Figure 4G–4H). And at the same time, the mean tumor weight from the xenograft mice was 0.54 ± 0.11 g after cell injection for 30 days, which was almost 2.8-fold heavier than the control group with 0.19 ± 0.11 g (*P* < 0.05, Figure 4I). These results showed that STC2 significantly promoted CRC development in mouse xenograft models.

### STC2 activates EMT process *via* AKT and ERK signaling pathways

**Figure 5:**
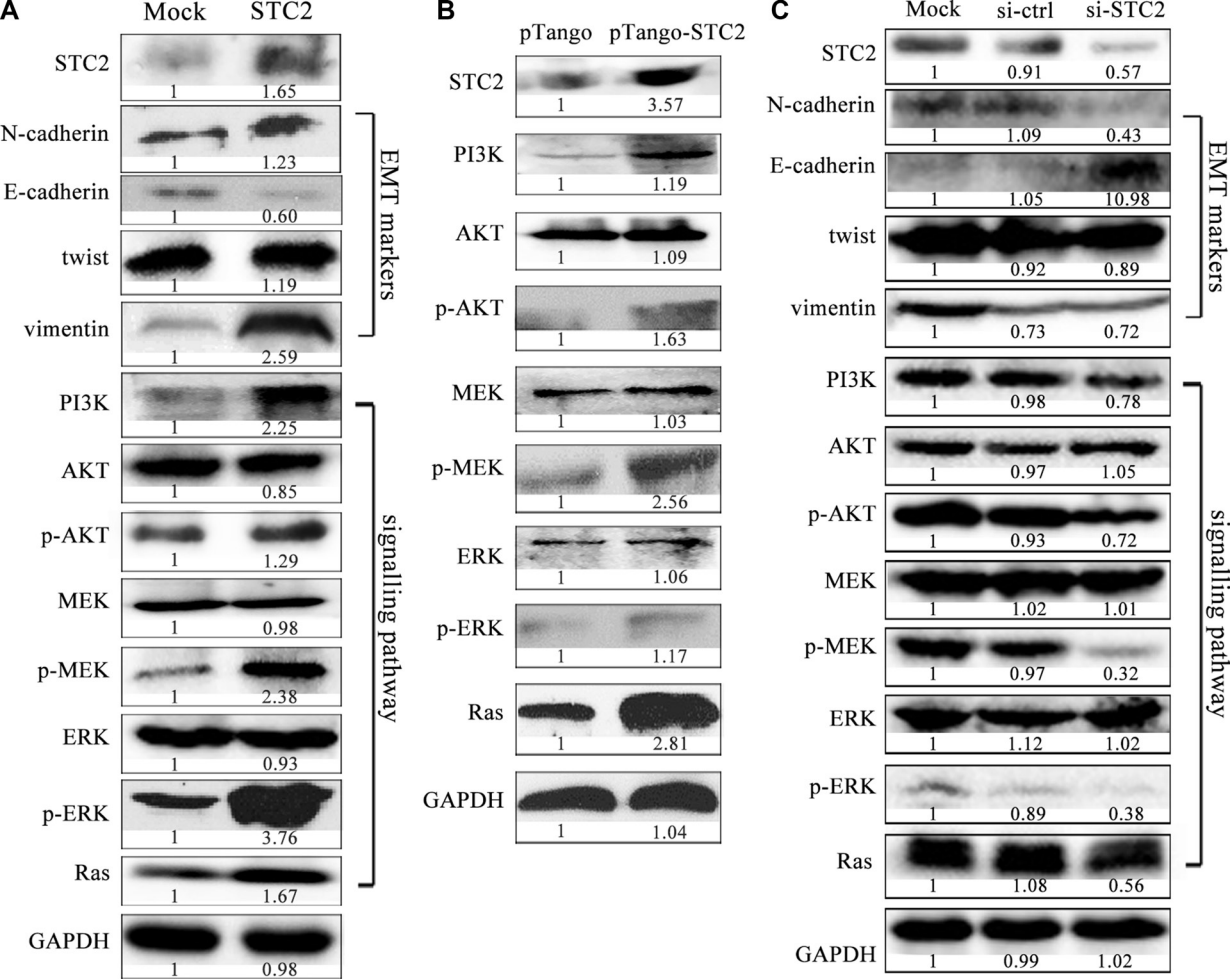
STC2 promotes EMT process *via* AKT and ERK signaling pathways EMT markers and the related PI3K/AKT, MEK/ERK signaling molecules were significantly changed in NCM460 cells incubated with the exogenous STC2 protein (STC2) (**A**) and transiently transfected with pTango-STC2 plasmids (pTango-STC2) (**B**). Conversely EMT markers and PI3K/AKT, MEK/ERK signaling molecules were oppositely changed in EMT cells treated with STC2 siRNA (si-STC2) for 48 h (**C**).

To clarify STC2-mediated molecular signaling pathways, exogenous STC2 protein (ab156715, Abcam) was directly added to incubate with NCM460 cells for 24 h to observe its effects. Similar as the result of CM treatment, NCM460 cells also obtained EMT morphology. More importantly, the expression of EMT biomarkers, such as N-cadherin, vimentin and twist, were dramatically increased, but E-cadherin was obviously decreased under STC2 protein induction (Figure 5A). Meanwhile, under STC2 stimulation, the phosphorylation of ERK, MEK and AKT was significantly increased, which has been reported to involve in EMT process [20–23]. And the upstream proteins, Ras and PI3K in MEK/ERK, AKT signaling pathways were upregulated too.

To avoid molecular level changes due to direct physical interference by the supplement of the exogenous STC2 protein, we also transfected STC2 plasmids into NCM460 cells to verify STC2-mediated signaling pathways. In pTango-STC2 expressing NCM460 cells, the phosphorylation of AKT, MEK and ERK were greatly increased too (Figure 5B). Meanwhile, the expression of PI3K and Ras was also markedly elevated in STC2-transfected cells. On the other hand, when the endogenous STC2 expression was inhibited by STC2-specific siRNA in EMT cells, EMT biomarkers and PI3K/AKT, Ras/MEK/ERK signaling molecules were changed oppositely (Figure 5C).

**Figure 6:**
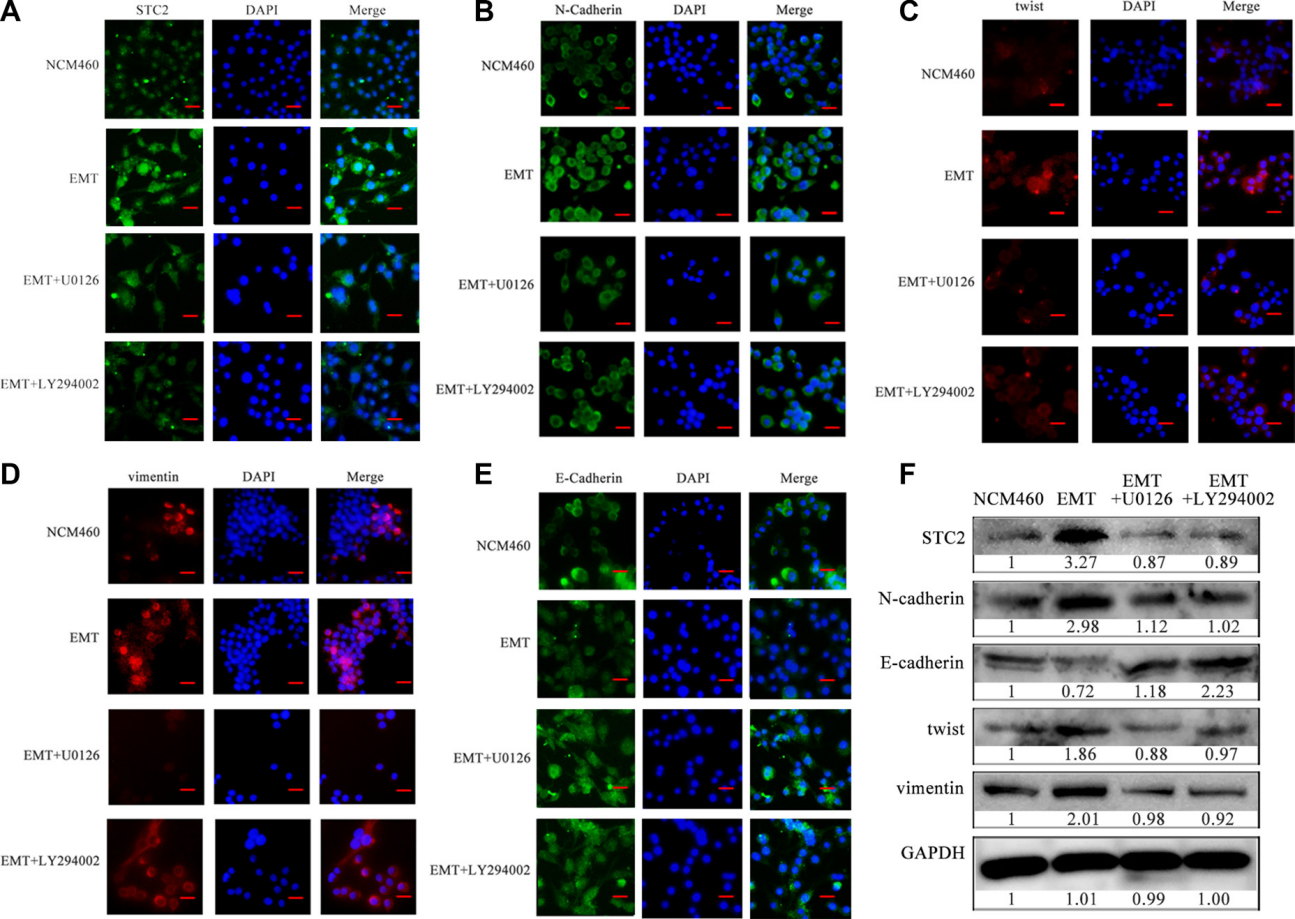
STC2 and EMT markers were altered in NCM460 and EMT cells by blocking AKT-ERK signaling pathways Proteins were observed through cell immunofluorescence staining (**A–E**) and Western blot (**F**). U0126: ERK inhibitor, LY294002: PI3K inhibitor. The scale bar represents 50 μm, with 100× microscopy image.

In addition, through blocking the AKT or ERK signaling with their inhibitors, STC2 and other EMT biomarker molecules were confirmed to change in EMT cells by cell immunofluorescence (Figure 6). Compared with the stronger endogenous fluorescence expression of STC2 in EMT cells (Figure 6A), STC2 expression became much weaker in EMT cells incubated with 10 μM ERK inhibitor U0126 or 10 μM PI3K inhibitor LY294002. Corresponding to the inhibitor incubation, the expression of N-cadherin, vimentin and twist, which were originally increased in EMT cells than NCM460 cells, was decreased in the inhibitor-treated EMT cells (Figure 6B–6D, 6F). Whereas the expression of E-cadherin showed an increase after inhibitor addition (Figure 6E, 6F). These results indicated that STC2 markedly stimulates NCM460 cell changes from epithelial to mesenchymal transition both in cell phenotype and in EMT biomarker expression through activating PI3K/AKT, Ras/MEK/ERK signaling pathways.

### Elevated STC2 expression and AKT/ERK phosphorylation in CRC tissues

**Figure 7:**
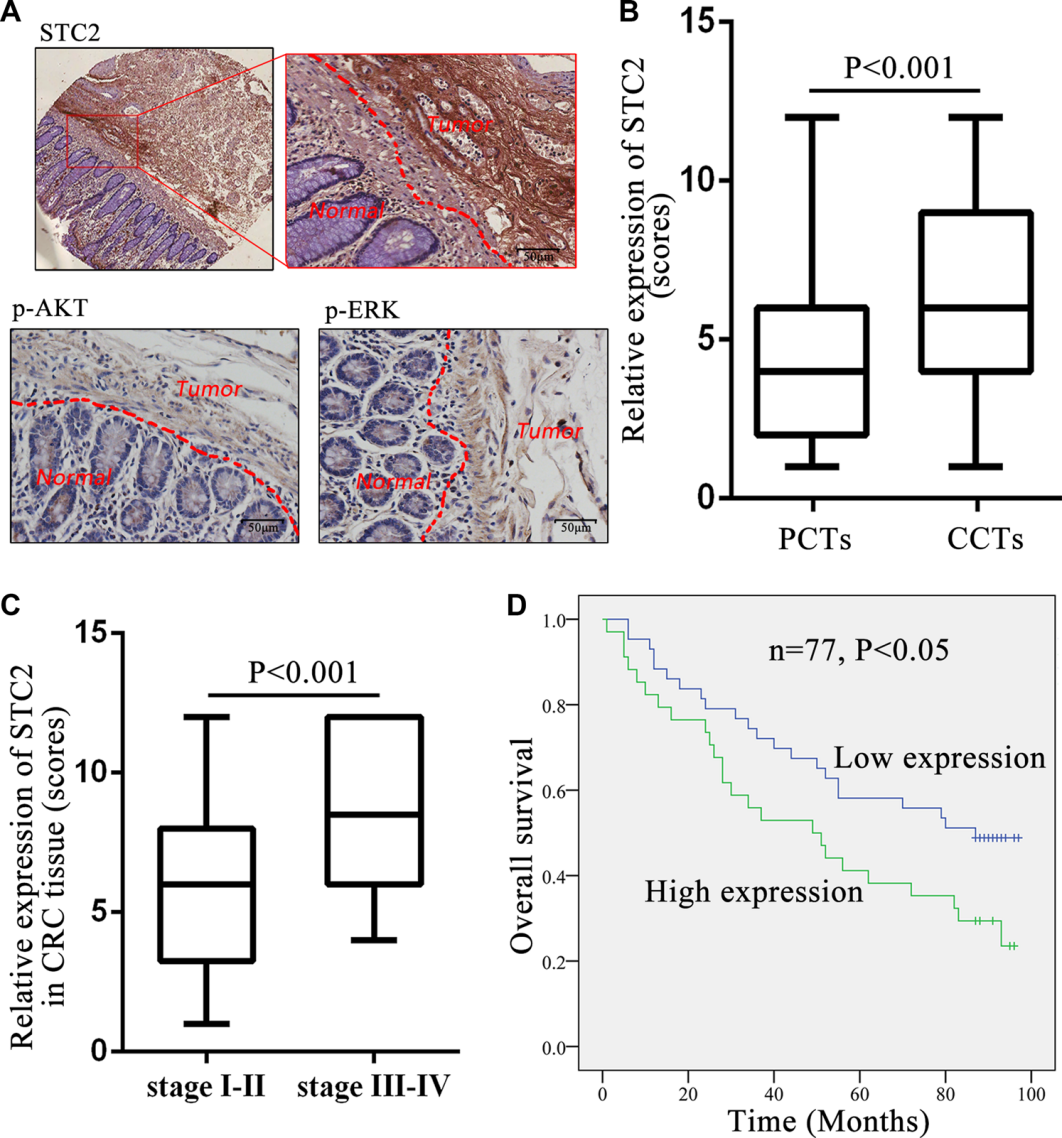
STC2 expression level in colorectal cancer tissues correlated with tumor stage and patient survival (**A**) Full image (up left) together with the snapshots (up right) of the STC2 immunostaining in colorectal cancer tissues (CCTs) and para-cancer tissues (PCTs). Representative images of the p-AKT and p-ERK immunostaining in CCTs and PCTs. The scale bar represents 50 μm, with 400× microscopy image. (**B**) STC2 expression was greatly increased in CCTs than PCTs (*n* = 77, *P* < 0.001). (**C**) Comparison of STC2 expression level in colorectal tumors with different TNM stages (stage I-II versus stage III-IV). (**D**) Kaplan-Meier overall survival curves for CRC patients based on STC2 expression. The overall survival rate for patients in STC2 low-expression group was significantly higher than the high-expression group (*P* < 0.05).

The elevated level of STC2 was further validated among 77 cases of human colorectal cancer tissues (CCTs) compared with patients' autologous para-cancer colorectal mucosa tissues (PCTs) by IHC (Figure 7A). The IHC staining intensity from weak, moderate and strong level for CCTs and PCTS was representatively shown in Supplementary Figure S4. And the tissue IHC scoring was summarized in detail in Supplementary Table S1. The average STC2 level with scoring 6.83 ± 3.40 was widely significantly increased in CCTs than a mean scoring 3.73 ± 2.44 in PCTs (Table 1 & Figure 7B, *P* < 0.001). Especially STC2 was highly expressed (overexpressed) in 44.16% CCTs with average scores 10.12 ± 1.83, which was more than the overexpression percentage (9.09%) in PCTs with average score 9.29 ± 1.25 (Table 1). Among 77 pairs of samples, over 90% PCTs (70 cases) showed low STC2 expression with average scores 3.17 ± 1.73, while about half of CCTs (55.84%) exhibited low STC2 expression with average scores 4.23 ± 1.60 (Table 1). And the expression distribution of STC2 in CCTs was located in cytoplasm and cell membrane.

**Table 1:** The immunoreactivity of STC2 between CCTs and PCTs

Immuno-reactivity	CCTs^1^ (*n* = 77)			PCTs^2^ (*n* = 77)			*P* value^3^
	Percentage	Total score	Average score	Percentage	Total score	Average score	
**Total**	100% (77/77)	526	6.83 ± 3.40	100% (77/77)	287	3.73 ± 2.44	< 0.001
**Low^4^**	55.84% (43/77)	182	4.23 ± 1.60	90.91% (70/77)	222	3.17 ± 1.73	
**High^4^**	44.16% (34/77)	344	10.12 ± 1.83	9.09% (7/77)	65	9.29 ± 1.25	

1 CCTs: colorectal cancer tissues;

2 CTs: para-cancer colorectal tissues;

3 The immunoreactivity differences between CCTs and PCTs groups were estimated using Student's *t*-test;

4 The low STC2 level was scored 1-6, while the high level was more than 6 scores.

Except for STC2, the STC2-mediated signaling molecules, pAKT and pERK, were also obviously increased in CCTs than PCTs (Figure 7A). These results indicated STC2 level and its regulated AKT and ERK signaling pathways were activated in CRC tissues.

### High STC2 level correlates with tumor stage and poor survival for CRC

The STC2 expression level (low expression versus high expression) was further shown to correlate with cancer clinicopathological information of CRC patients. High STC2 expression was positively correlated with high-grade tumorigenesis stage for CRC, including tumor size, lymph node metastasis and TNM stage (*P* < 0.05) (Table 2). The average expression of STC2 in CCTs with TNM III-IV stages (8.81 ± 3.19) had almost 1.5-folds of those with TNM I-II stages (5.82 ± 3.06) (*P* < 0.001, Figure 7C). Furthermore, the incidence of lymph node metastasis was higher in the STC2 high-expressing CCTs (52.9%, 18/34) than the low-expression tissues (18.6%, 8/43) (*P* < 0.001). However, STC2 expression level had no significant correlations with patient's gender and age (*P* > 0.05).

**Table 2:** Correlation between STC2 level and the clinicopathological parameters of CRC specimens

Clinicopathological parameters	All patient Number (*n*)	Expression level of STC2 (*n* = 77)		
		Low^1^ Number (*n*)	High^1^ Number (*n*)	*P* value^2^
**Total**	77	43	34	
**Sex**				
Male	40	23	17	0.538
Female	37	20	17	
**Age (years)**				
< 71	35	21	14	0.799
≥ 71	42	22	20	
**Tumor size**				
< 3 cm	21	16	5	0.004
≥ 3 cm	56	27	29	
**Lymph node metastasis**				
N0	51	35	16	0.001
N1–N2	26	8	18	
**TNM stage**				
I–II	51	35	16	< 0.001
III–IV	26	8	18	

1 The STC2 low-expression group was averagely scored 4.27 ± 1.61, while the high-expression group was averagely scored 10.05 ± 1.82.

2 Differences between groups were estimated using x^2^ test.

We further found that the level of STC2 in CCTs negatively correlated with the overall survival rate of CRC patients. The Kaplan–Meier analysis indicated the median overall survival among patients with low STC2 expression in tumor tissues was 59.96 ± 31.68 months as compared with 54.50 ± 34.10 months among those with high expression (*P* < 0.05, Figure 7D). The association between the higher STC2 expression and poorer overall survival was significant, which was concluded from a multivariable analysis using the Cox proportional hazards model (hazard ratio [HR] = 0.677; 95% confidence interval [CI] = 0.368– 1.248; *P* = 0.042).

### Serum STC2 level correlates with CRC progression and prognosis

**Figure 8:**
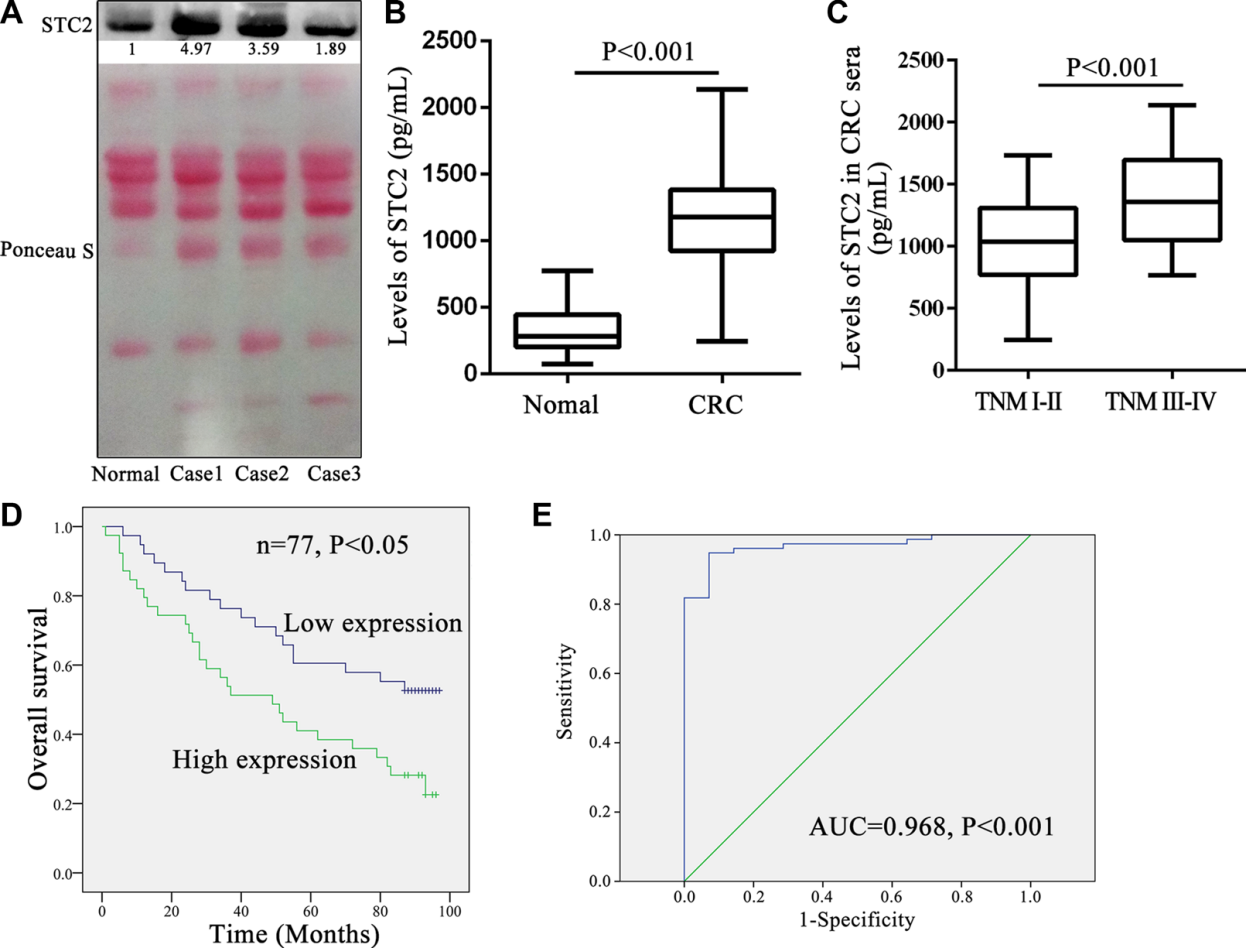
Serum STC2 level correlated with tumor stage and overall survival for colorectal cancer patients (**A**) The secretion levels of serum STC2 were compared between colorectal cancer (CRC) patients and normal person by western blot. Normal: sera from the health donor; Case1-3: 3 cases of CRC sera. Reversible Ponceau staining was used as a loading control. (**B**) The mean serum STC2 level of CRC patients (*n* = 77) was much higher than health donors (*n* = 14) (*P* < 0.001). (**C**) Comparison of serum STC2 level in CRC patients with TNM stage I-II versus those with stage III–IV. (**D**) Kaplan-Meier overall survival curves for CRC patients based on serum STC2 levels. The overall survival rate for patients with low serum STC2 level was significantly higher than those of patients with high serum STC2 level (*P* < 0.05). The low serum STC2 level was defined as less than 1179.35 pg/mL (median value) (average 834.92 ± 242.55 pg/mL), while the high serum STC2 level was above 1179.35 pg/mL (average 1463.61 ± 241.70 pg/mL). (**E**) A ROC analysis was performed based on the ELISA results. Empirical ROC curves deviated from typical ROC-curve shapes, and a portion of the curve leading to the northeast corner of the ROC space had relatively steep and constant slopes. AUC: area under the ROC curve.

Due to STC2 secretion in extracellular environment *in vitro*, human serum STC2 level was more conveniently monitored than tissue samples for CRC patients by Western blot and ELISA. Three cases of CRC serum detection by Western blot showed STC2 secretion was obviously elevated in CRC patients than healthy donors (Figure 8A). And it was consistent that the mean 1153.35 ± 397.42 pg/ml serum STC2 in CRC patients was 3.5-folds of 331.08 ± 184.17 pg/ml level in healthy persons (*P* < 0.001, Figure 8B).

Similarly the serum STC2 level well correlated with tumor size, lymph node metastasis, and TNM stage of CRC (*P* < 0.05) (Table 3), which was consistent with the conclusion from tissue STC2 level. The serum concentration of 1382.05 ± 373.70 pg/ml STC2 in CRC with TNM stage III-IV was significantly increased than that 1036.76 ± 359.42 pg/ml level for TNM stage I–II (*P* < 0.001, Figure 8C). Meanwhile the median overall survival among patients with low serum STC2 level was 64.15 ± 30.61 months as compared with 50.56 ± 33.76 months among those with high serum STC2 level (*P* < 0.05, Figure 8D). Therefore the serum STC2 level correlated inversely with prognosis of CRC patients, and patients with high serum STC2 level had poor postoperative overall survival. Moreover, based on the serum STC2 ELISA detection, ROC curve describing the discrimination of patients diagnosed with CRC (*n* = 77) from healthy people (*n* = 14) with STC2 levels was shown in Figure 8E. The area under the ROC curve was 0.968 (*P* < 0.001), which indicated that serum STC2 is sensitive and specific to differentiate healthy individuals and cancer patients.

**Table 3:** Correlation between serum STC2 levels and the clinicopathological parameters of CRC specimens

Clinicopathological parameters	Patient Number (*n*)	Serum STC2 level (*n* = 77)		
		Low^1^ Number (*n*)	High^1^ Number (*n*)	*P* value^2^
**Total**	77	43	34	
**Sex**				
Male	40	20	20	0.727
Female	37	18	19	
**Age (years)**				
< 71	35	21	14	0.164
≥ 71	42	17	25	
**Tumor size**				
< 3 cm	21	14	7	< 0.001
≥ 3 cm	56	24	32	
**Lymph node metastasis**				
N0	51	29	22	0.002
N1–N2	26	9	17	
**TNM stage**				
I–II	51	29	22	< 0.001
III–IV	26	9	17	

1 The median serum STC2 level was 1179.35 pg/mL in CRC patients, which was taken as the cut-off value to define low and high serum STC2 level. The low serum STC2 level was 834.92 ± 242.55 pg/mL, and high serum STC2 level was 1463.61 ± 241.70 pg/mL.

2 Differences between groups were estimated using Χ^2^ test.

## DISCUSSION

The expression level of STC2 is dramatically increased in several cancers, including colon cancer, gastric cancer, prostate cancer and renal cancer [8–11]. And STC2 also involves in ovarian cancer cell invasiveness activity [24]. But the molecular mechanism of STC2 on cancer cell behaviors is still poorly discovered. In this study, our findings indicate STC2 not only activates normal colon mucosal epithelial cells to acquire EMT features, and it also promotes colon cancer cell invasion and migration via ERK/MEK and PI3K/AKT signaling pathways. Meanwhile the elevated STC2 level and its clinic correlations widely exist in CRC tissues and patient serum samples. Therefore STC2 is a novel tumor biomarker for CRC.

The concept of EMT was originally defined as a morphological conversion during embryogenesis occurring at specific sites in embryonic epithelia to give rise to individual migratory cells [13, 25]. More and more researches indicate EMT is a phenotypic conversion linked with cancer metastasis [25–28]. And morphological features of EMT have been mostly reported in human cancers of epithelial origin [29]. In colon cancer, the EMT has been demonstrated to be a dominant program [16, 30].

The previous studies on EMT mainly focused on cancer metastasis profiles. In fact, the progression from normal intestinal mucosa to adenoma (adenomatous mucosa) and finally to adenocarcinoma in CRC is closely correlated with the EMT process and changes in the expression of a series of genes, such as E-cadherin, vimentin, and β-catenin [31, 32]. So we first investigated the original changes of EMT-like process on human colon mucosal epithelial cells repeatedly induced by a chemical reagent PMA. PMA as an analogue to diacylglycerol could activate protein kinase C in cytoplasm and lead to malignant of cancer [24]. In our study, PMA was used as an inducer to continuously stimulate NCM460 cells and finally make NCM460 cells acquire EMT characteristic step-by-step (Figure 1A, 1B). It is noted that different cell lines have different tolerance ability to PMA. For example, human prostate cancer ARCaPE cells were treated with 1000 nM PMA to induce EMT [17]. In our experiment, cell vitality was poor as over 300 ng/ml PMA incubation with NCM460 cells, and 500 ng/ml PMA in the medium induced cell death. Therefore we selected 100 ng/ml PMA to induce NCM460 cells for EMT establishment. After stimulation with PMA for 24 hours, NCM460 cells gradually exhibited some mesenchymal features, but EMT biomarkers significantly changed since NCM460 cells were induced with PMA for eight generations of cell passages (Figure 1B). At this original period with PMA treatment for 24 h, if the EMT-like NCM460 cells were terminated to incubate with PMA for another 24 h, the spindle-shaped cells would reverse back to epithelia states, from spindle to cobblestone-like shapes, and the expression level of EMT biomarker also changed responsive to cell morphology. So, the transition of NCM460 cells to EMT was a progressive and continuous process. Finally the epithelial NCM460 cells were stimulated by PMA to gain mesenchymal properties with lower intercellular adhesion and higher motility (Figure 1C, 1D).

Due to protein expression changes in EMT cells, conversely what do secreted proteins derived from EMT cells have effects on normal epithelial cells and colorectal cancer cells? We collected total serum-free cell supernatant secreted by PAM-induced EMT cells to observe its effects on normal NCM460 and colon cancer HT29 cells. As expected, cell morphology and EMT-related proteins were both changed in NCM460 and colon cancer HT29 cells which were incubated with CM from EMT cells (Figure 2). Furthermore we verified the STC2 protein level contributed to EMT development *in vitro* and *in vivo*.

**Figure 9:**
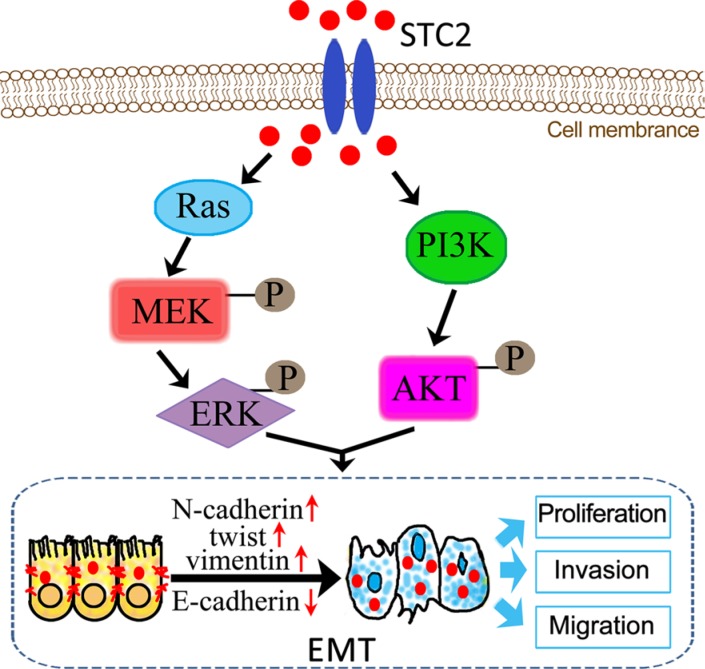
Schematic illustration of STC2-promoted EMT process through activation of AKT and ERK signaling pathways

EMT is mediated by several different signaling pathways [33]. Because AKT/ERK signaling pathways widely participate in cell proliferation, differentiation and metabolism, we investigated the STC2-mediated AKT/ERK molecular signaling pathways in EMT process. In our study, in response to EMT molecular marker changes, the expression of Ras, PI3K, p-AKT, p-MEK and p-ERK was highly increased in STC2-overexpressing NCM460 cells either by transient transfection of STC2 plasmids or by exogenous STC2 protein induction (Figure 5A, 5B), which indicated STC2 could activate the AKT and ERK signaling pathways. Conversely STC2 knockdown in EMT cells caused reverse changes of EMT markers and the AKT-ERK signaling molecules (Figure 5C). Furthermore by blocking the PI3K/AKT or MEK/ERK signaling with their inhibitors, LY294002 and U0126, the STC2 and other EMT biomarker molecules were also validated to alter in EMT cells (Figure 6). The results suggest STC2 activates EMT process via two signaling pathways including PI3K/AKT and MEK/ERK in colorectal cancer cells. Our data are consistent with that previous conclusion which reported STC2 activates ERK levels to enhance ovarian cancer EMT and invasiveness [23]. We summarized STC2-mediated signaling pathways to involve in cancer EMT process (Figure 9), which is a pivotal step for colorectal cancer metastasis [16, 33].

Additionally, the clinical colon cancer tissues and patient serum analysis were further supported that higher level of STC2 protein is present in colorectal tumor and patient's serum compared with normal samples, which also has linkage with tumor pathologic classification and CRC patient survival. It is noticed that STC2 is a secreted protein, STC2 secretion level into CRC patient plasma is at least 3 folds of healthy person. The secreted level of STC2 in CRC patient's plasma can really reflect cancer progression status including lymph node metastasis, tumor TNM stages and overall survival, which was completely consistent with correlations of tissue STC2 expression and clinical significance. Therefore the plasma STC2 level is a promising serum biomarker for CRC diagnosis and prognosis.

Extracellular factors including cytokines and proteins could regulate EMT process by multiple receptor mediated signaling pathways, such as transformation growth factor receptor and tyrosine kinase receptors, which accept signal came from epidermal growth factor and filamentary growth factor etc. IL-8 could also be used as a stimulating factor and combined with IL8R that located on cell membrane to promote tumor cell occurrence of EMT [34]. By now, the binding receptor of STC2 protein is not clear. Although STC2 was found to co-locate with estrogen receptor and progesterone receptor on cell membrane [35, 36]. By our bioinformatics analysis using the online software STRING, tyrosine kinase receptor was predicted as a potential receptor of STC2 (data not shown). The STC2 expression can cause Ras level change (Figure 5), which indicates STC2 seems to link with tyrosine kinase receptors including Ras and Raf in EMT. Meanwhile our data suggest that STC2 could impact adjacent epithelia or cancer cells by a paracrine way (Figure 3C–3D). Our findings support STC2-mediated EMT process is associated with colon cancer occurrence and development, which gives a new insight on biotherapy of colon cancer by preventing progression of EMT through silencing STC2 expression.

In conclusion, we elucidated the linkage of STC2 with colon cancer development *in vitro* and *in vivo*. STC2 promotes occurrence and migration of colorectal cancer via the EMT process, because the elevated STC2 level and its clinicopathologic correlations widely exist in CRC tissues and serum samples. All these findings suggest STC2 is a potential tumor biomarker and a serum biomarker for CRC diagnosis and prognosis.

## MATERIALS AND METHODS

### Cell culture

The NCM460, a normal human colon mucosal epithelial cell line [37], was ordered from Incell Corporation, LLC (www.incell.com). HT29, a colon adenocarcinoma cell line, were purchased from American Type Culture Collection. Cells were routinely maintained in Dulbecco's Modified Eagle's Medium (DMEM) (Gibco, Gaithersburg, MD) containing 10% fetal bovine serum (FBS) (16000–044, Gibco, USA) at 37°C in humidified atmosphere with 5% (v/v) CO_2_. However cells were cultured in serum-free conditioned media (CM) to detect secreted proteins.

To modulate cell interactions *in vitro* between two kinds of cell lines, HT29 and NCM460 cells were co- cultured with CM based on a transwell insert system [15]. In this system, NCM460 cells, seeded in 6-well cell culture inserts (Millipore, PIHT30R48), were co-cultured with HT29 cells in a 6-well plate at 1:1 cell number ratio. After co-cultured in serum-free DMEM media for 48 h, cell medium supernatant was respectively collected from mono-cultured NCM460, mono-cultured HT29 cells, and the co-cultured NCM460:HT29 cells for analysis. Correspondingly, cell pellets were respectively collected from the mono-cultured NCM460, HT29 cells, the NCM460 cells co-cultured with HT29 cells (TAE), and the co-cultured NCM460:HT29 cells together to detect EMT molecular expression according to our previous methods [15].

### EMT was induced from colorectal cells by PMA stimulation

The NCM460 cells were induced with a chemical reagent, 100 ng/ml phorbol-12-myristate-13-acetate (PMA) [17]. Cell passage was performed after cell growth for 2 days. PMA was added to incubate with cells at the time point of 12 h after cell passage. Cell phenotypes were observed, and EMT markers were detected in NCM460 cells incubated with 100 ng/ml PMA respectively for 0, 3, 7, 10, and 14 days, approximately 0, 1, 3, 5, 7 cell passages. Therefore the EMT cells were acquired from stable cell clones with EMT features after a continuous PMA stimulation for 8 cell passages.

### Cell incubation with EMT cell-derived conditioned media

EMT cells, which were induced by PMA for 8 generations, were collected to wash 3 times with PBS to eliminate PMA remnant in culture media. Then EMT cells with no PMA were further cultured in serum-free medium for 24 h to collect cell supernatants (conditioned media, CM) by 4000 g centrifugation. A series of different amount of EMT cell-derived CM (from 0.2, 05, 1.0 to 1.5 ml) was respectively added to incubate with NCM460 or HT29 cells for 24 hours, which were seeded into a 6-well plate with a final volume of 2 mL culture media. Then EMT markers were detected in these CM-treated NCM460 and HT29 cells.

### Colon cancer tissues and serum samples

All following manipulations were performed in full accordance with guidelines of the West China Hospital Medical Center Institutional Review Board of Sichuan University. And the patients recruited had a written consent to participate this research. Human colorectal cancer tissues (CCTs) (*n* = 77) and patients' autologous para-cancer normal colorectal tissues (PCTs) were collected from CRC patients who underwent surgery at West China Hospital, Sichuan University (Chengdu, P. R. China). Each case was identified through pathologic biopsy. All patients were followed up until August 2015 with a median observation time of 62 months. The clinical characteristics of the research subjects were shown in Supplementary Table S2.

In order to analyze the serum STC2 level, 5 ml of peripheral blood samples for each person was respectively collected from 77 colon cancer patients before surgery. We also got plasma sample from 14 healthy donors to detect STC2 level, which was used as normal control. All serum samples were processed according to our previous procedures to minimize pre-analytical variation [38]. Serum was then frozen immediately at −80°C for use.

### Plasmid construction and cell transfection

The amplified STC2 cDNA and expression vector pTango were doubly digested with restriction enzymes *BamH* I and *Xho* I respectively, and were ligated overnight at 16°C and transformed into competent *E. coli* DH5α cells. The transformation mixture was plated on to LB agar containing 100 μg/ml ampicillin to incubate for 16 h at 37°C to pick up recombinant plasmids. The recombinant plasmid pTango-STC2 was confirmed by restriction enzyme digestion with *Bam*HI*/Xho* I and DNA sequencing.

For STC2 exogenous overexpression, the pTango-STC2 plasmids and control empty vector were separately transfected into NCM460 cells using Lipofectamine 2000 reagent (Invitrogen, Carlsbad, CA, USA).

The siRNA-mediated STC2 knockdown was performed by transfecting the synthetic siRNA duplexes for 24–96 h. The siRNA sequences were designed as follows: 5′-CAGCGGAGAUCCAGCACUGdTdT-3′ (sense), 5′-CA GUGCUGGAUCUCCGCUGdTdT-3′ (antisense). The siR-Ribo negative control (siN05815122147–1-2, RiboBio, Guangzhou, China), with no significant sequence homology to mammalian genes, was used as the control.

### ELISA assay

The cellular secretion level of STC2 in cell conditioned media from NCM460, HT29 and EMT cells, as well as human serum STC2 was measured by the enzyme-linked immunosorbent assay (ELISA). The ELISA procedures were mainly followed our previous methods [15]. The CM or serum samples were added into a 96-well plate, which was pre-coated with anti-STC2 antibodies (sc-14350, Santa Cruz, USA), to incubate for 1 h at 37°C. After washing 5 times with PBST (3.2 mM Na_2_HPO_4_, 0.5 mM KH_2_PO_4_, 1.3 mM KCl, 135 mM NaCl, 0.05% Tween 20, pH 7.4), the secondary antibody was added into the plate to incubate 1 h at 37°C. Finally then reactions were stopped with 1N HCl, and ELISA plates were detected using an ELISA Reader (Multiskan Mk3, thermo) at 450 nm, with correction at 570 nm.

### Western blot

Several target proteins were detected their expression by Western blot. Proteins were separated on a 12% SDS-PAGE gel and transferred onto a PVDF membrane at 100 V for 1 h. The specific primary antibodies, including vimentin (1:500, sc373717, Santa Cruz), E-cadherin (1:500, sc-31021, Santa Cruz), N-cadherin (1:500, sc-31031, Santa Cruz), twist (1:500, sc-81417, Santa Cruz), STC2 (1:500, sc-14350, Santa Cruz), ERK (1:500, sc-292838, Santa Cruz), MEK (1:500, sc-436, Santa Cruz), p-ERK (1:500, sc- 136521, Santa Cruz), p-MEK (1:1000, 9121, Cell signaling), Akt (1:1000, 4961, Cell signaling) and phospho Akt (1:1000, 2118–1, Epitomics), were diluted in TBST buffer (50 mM Tris–HCl, with 150 mM NaCl, 0.1% Tween-20, pH 7.4) to incubate PVDF membrane at 4°C overnight. The corresponding secondary antibodies, conjugated horseradish peroxidase, were subsequently incubated with the PVDF membrane at 37°C for 1 h. Signal detection was performed with HRP substrates (WBLUR0100, Millipore). The detection of GAPDH against its antibody (1:1000, sc- 365062, Santa Cruz) was taken as a control.

For serum STC2 detection, the high abundant serum albumin and IgG was removed by using a reagent kit (ProteoExtract Albumin/IgG Removal Kit, 122642, Calbiochem, San Diego, CA) [38]. Reversible Ponceau staining was used as a loading control.

### Cell viability

Cell viability was measured using MTT assay. After STC2 overexpression or knockdown for 24 h, 5 × 10^3^ HT29 cells/well were seeded in one 96-well plate in DMEM supplemented with 10% FBS to incubate for 12, 24, 48, 72, 96 h. At each time point, 20 ul of MTT solution (5 mg/ml, Sigma) was added to each well to incubate for 2–4 h at 37°C, the formazan crystals were dissolved with 150 ul dimethyl sulfoxide (Sigma). Absorbance was determined at 570 nm on Multiskan MK3 (Thermo Scientific) immediately.

### Cell invasion and migration assay

Cell invasion and migration ability was detected with a transwell system based on a transwell 24-well chambers (Millipore) as described before [39]. After being transfected with pTango-STC2 plasmids or STC2-specific siRNA for 48 h, 1 × 10^4^ cells in 200 ul serum-free DMEM were seeded in the upper chamber of a transwell, and the bottom of the chamber was filled with 600 μl of DMEM containing 10% FBS. For invasion assay, matrigel (1:4, BD, USA) was added to the transwell chambers previously and incubated at 37°C for 4 h. Cells on the upper side of the filter were removed after 24 h for the migration assay or 48 h for the invasion assay. The filter membrane was stained with crystal violet, and the number of the cells that remained adherent to the underside of the membrane were counted using an inverted microscope (Zeiss Axiovert).

### Immunofluorescence analysis

The expression of STC2 and EMT markers was observed in cultured EMT cells by an immunofluorescence microscopy. Cells grown on coverslips, which were performed in a 96-well plate, were fixed with paraformaldehyde for 30 min. After fixation, cells were washed with TBST twice and blocked in 1.5% BSA/TBST for 1 h at room temperature. Cells were respectively incubated with primary antibodies against STC2 (1:500, sc-14350, Santa Cruz), vimentin (1:500, sc373717, Santa Cruz), E-cadherin (1:500, sc-31021, Santa Cruz), N-cadherin (1:500, sc-31031, Santa Cruz) and twist (1:500, sc-81417, Santa Cruz) overnight at 4°C. Then cells were incubated with TRITC-conjugated secondary antibodies (ZF-0313, ZSGB-BIO) and FITC-conjugated ones (ZF-0314, ZSGB-BIO) for 1 h and stained with DAPI for 5 min. The images were viewed and recorded by Olympus BX40 and SPOT Flex (Diagnostic Instruments, Version 4.5).

To block ERK, PI3K signaling, 10 μM ERK inhibitor U0126 (#S1901, Beyotime) or 10 μM PI3K inhibitor LY294002 (#9901, Cell Signaling Technology) was respectively incubated with NCM460, EMT cells for 24 h to directly observe expression strength of STC2 and EMT markers. Other procedures were same as the above.

### Immunohistochemistry

The relative protein expression from tissue samples was analyzed by immunohistochemistry (IHC). The IHC manipulations were performed mainly according to our previous procedures [39, 40]. Generally, the paraffin-embedded tissues were cut into slices with 4-μm thickness to dewax and rehydrate with Gradient ethanol. After quenching the endogenous peroxidase activity and antigen retrieval, tissue sections were respectively incubated with a primary antibody, including anti-STC2 (sc-14350, Santa Cruz, USA), anti-pERK (Santa Cruz, USA), anti-pAKT (Epitomics, USA) at 4°C over night. Then slides were incubated with horseradish peroxidase-linked secondary antibodies at 37°C for 40 min, followed by reaction with 3, 3′-diaminobenzidine substrate solution (Dako Cytomation GmbH) and counterstaining with Mayer's hematoxylin.

IHC analysis of tissue slices was evaluated by two pathologists in blinded fashion to minimize variability (Supplementary Table S1). Estimated percentage of staining was determined by calculating the average of stained cells from 3–4 microscopic fields under 20-fold magnification. According to general evaluation standards [24, 40], the percentage of positive stained cells was quantified into five groups: 0 to 5% positive cells for scoring 0; 5% to 25% positive cells for scoring 1; 25% to 50% positive cells for scoring 2; 50% to 75% positive cells for scoring 3 and ≥ 75% positive cells for scoring 4. And the intensity level was scored as 0 as absence of staining, 1 for weak, 2 for moderate, and 3 for strong staining. The multiplication of the percentage of positive staining and the intensity was used to define STC2 expression levels: negative (0), low expression (scores 1–6) and high expression (overexpression, scores > 6).

### Tumorigenicity assay in nude mice

All animal experiments were performed in accordance with the guidelines of Sichuan University and approved by the Animal Care Committee of Sichuan University (Chengdu, China). Male BALB/c-nude mice (4 weeks old), each weighing 18–20 g, were ordered from Vital River company (Beijing, China). The nude mice were housed in a specific-pathogen-free (SPF) environment, and handled in strict accordance with good animal practice.

Mice were randomly divided into two groups, and 6 mice were included for each group (*n* = 6). The experimental group was injected the stable pTango-STC2 transfected HT29 cells to observe tumor growth, and another control group was injected the empty vector pTango-containing HT29 cells. 1 × 10^7^ pTango-STC2 –containing HT29 cells in 0.1 mL serum-free DMEM were injected subcutaneously into the right flank of each mouse for the experimental group [41], and same quantity of pTango-containing HT29 cells was used in the control group. When palpable tumors arose, tumor growth was monitored by caliper measurements of the largest and perpendicular diameters every 3 days. Tumor size was calculated with the formula V = (width^2^×length/2) [42, 43]. The final data were averagely obtained from three replicate experiments. Finally all mice were killed to measure tumor weight after cell injection for 30 days.

### Statistical analysis

Statistical analysis was performed with SPSS 19.0 for Windows (SPSS Inc). The Pearson χ2 test was used to compare qualitative variables, and quantitative variables were analyzed by the Student *t* test (One-way ANOVA for differences among more than two groups). Kaplan-Meier analysis was used to analyze the patient survival. The Cox regression model was used to perform multivariate analysis. Receiver operating characteristic (ROC) curve analysis was used to determine the predictive value of the expression level of STC2. *P* < 0.05 was considered statistically significant.

## SUPPLEMENTARY MATERIALS FIGURES AND TABLES




