# Supplementary material for: STC2 promotes the epithelial-mesenchymal transition of colorectal cancer cells through AKT-ERK signaling pathways
Source: Oncotarget. 2016 Sep 20;7(44):71400–16. doi: 10.18632/oncotarget.12147 (PMC5342087; doi:10.18632/oncotarget.12147)
Supplement: Supplementary file 1 [file oncotarget-07-71400-s001.pdf]

STC2 promotes the epithelial-mesenchymal transition of colorectal cancer cells through AKT-ERK signaling pathways

Supplementary Materials

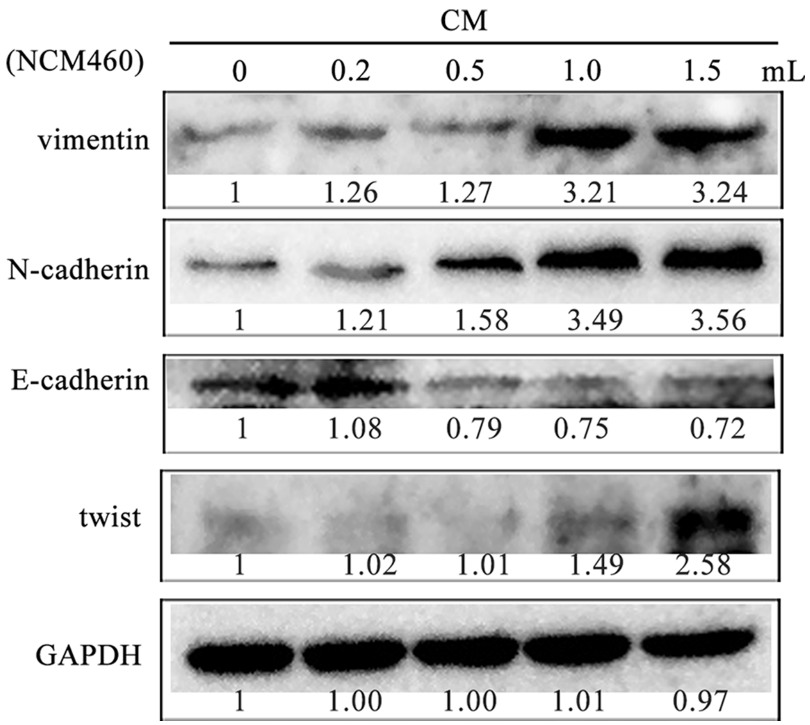

Supplementary Figure S1: EMT biomarker expression was detected in NCM460 cells incubated with EMT cell-derived CM, in which additional FBS was added into cell culture to ensure final 10% FBS in culture media. Actually the little volume of additional FBS almost had no influence on cell growth for 24 h, including EMT molecule expression. CM: conditioned media.

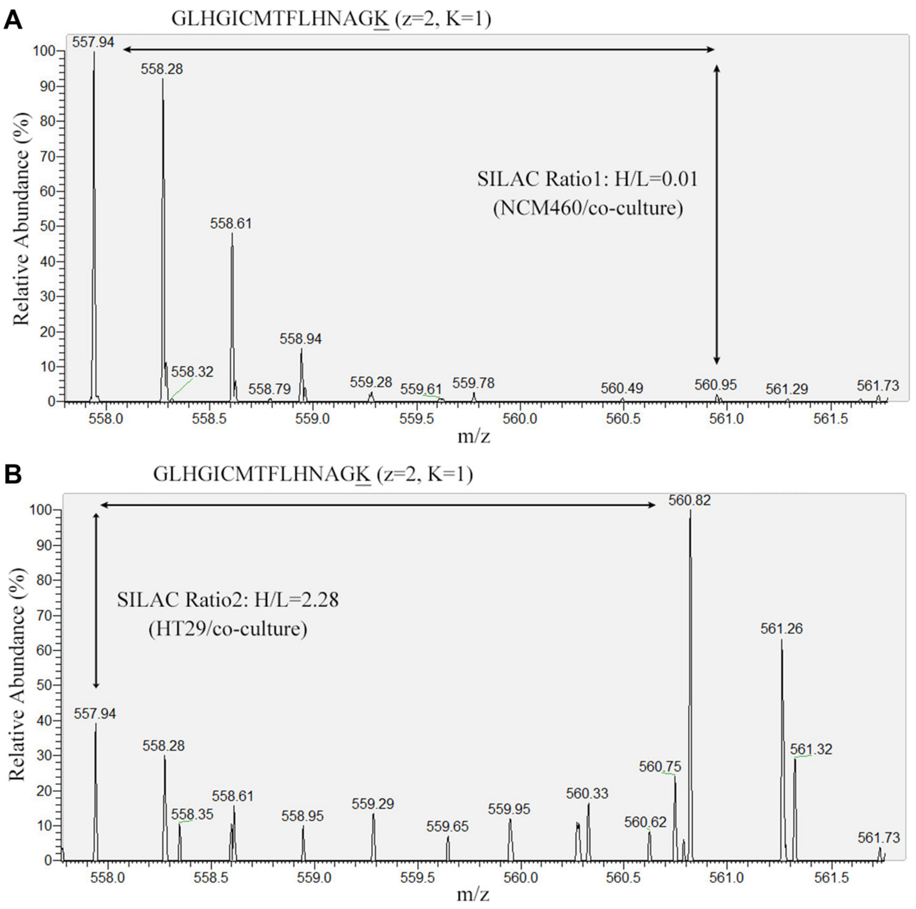

Supplementary Figure S2: Quantitative MS analysis for the secretion of STC2 in mono-cultured cells and co-cultured HT29: NCM460 cells. The SILAC ratio1 for sample 1 (mono-cultured NCN460 versus co-culture) and SILAC ratio2 of sample 2 (mono-cultured HT29 versus co-culture) were respectively calculated by peak intensity ratio of "labeled" (heavy) peptide versus "unlabeled" (light) one. (**A**) MS spectra of a unique isotope labeling peptide (m/z 557.94, m/z 560.95) "GLHGICMTFLHNAGK" of STC2 from sample 1. (**B**) MS spectra of a unique isotope labeling peptide (m/z 557.94, m/z 560.82) "GLHGICMTFLHNAGK" of STC2 from sample 2. Z was charge number; K was the abbreviation of $^{13}C_6$-Lys.

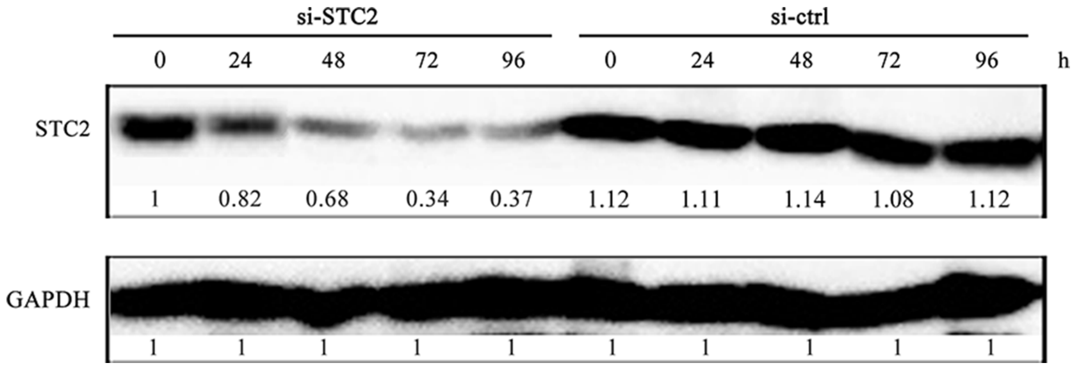

Supplementary Figure S3: STC2 knockdown analysis by STC2-specific siRNA for 24-96h. si-STC2: STC2-specific siRNA; si-ctrl: unspecific siRNA as control.

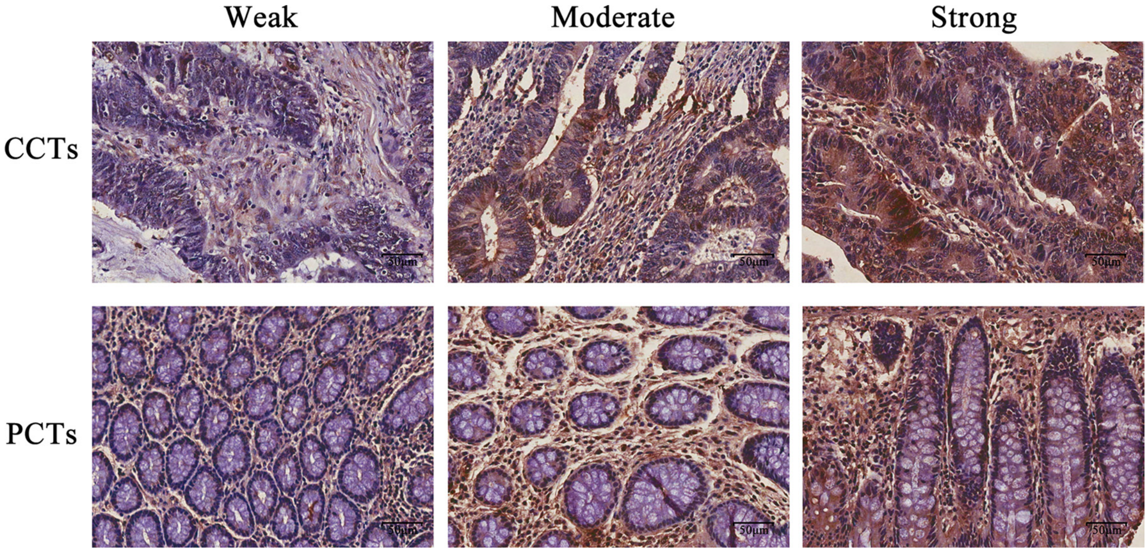

Supplementary Figure S4: A panel of representative tissues showing the weak, moderate and strong staining of STC2. The expression of STC2 in CRC tissues (CCTs) and patients' autologous para-cancer colorectal tissues (PCTs) was detected by immunohistochemistry, and the representative images showed the intensity levels of weak, moderate and strong staining in CCTs and PCTs. (400×, the scale bar represents 50 μm).

Supplementary Table S1: Tissue IHC scores for STC2 expression evaluated by two pathologists.
See Supplementary_Table_S1

Supplementary Table S2: Clinicopathaological characteristics of colorectal cancer cases

Characteristic	Number (N = 77)	%
**Sex**		
Male	40	51.95%
Female	37	48.05%
**Age (years)**		
< 71	35	45.45%
≥ 71	42	54.55%
**Primary tumor**		
T1	1	1.30%
T2	7	9.09%
T3	56	72.73%
T4	13	16.88%
**Lymph nodes metastasis**		
N0	51	66.23%
N1	19	24.68%
N2	7	9.09%
**Distant metastasis**		
M0	76	98.70%
M1	1	1.30%
**TNM stage***		
I	8	10.39%
II	43	55.84%
III	25	32.47%
IV	1	1.30%

*The criterion of the TNM stage is determined according to the American Joint Committee on the Cancer (AJCC) Cancer Staging Manual, Seventh Edition (2010).
