# Supplementary material for: STC2 promotes the epithelial-mesenchymal transition of colorectal cancer cells through AKT-ERK signaling pathways
Source: Oncotarget. 2016 Sep 20;7(44):71400–16. doi: 10.18632/oncotarget.12147 (PMC5342087; doi:10.18632/oncotarget.12147)
Supplement: Supplementary file 2 [file oncotarget-07-71400-s002.doc]

**Supplementary Table 1**. Tissue IHC scores for STC2 expression evaluated by two pathologists

|  | **Score 1** | | **Score 2** | | **Average score** | |
| --- | --- | --- | --- | --- | --- | --- |
| **Tissue number** | **cancer** | **para-cancer** | **cancer** | **para-cancer** | **cancer** | **para-cancer** |
| RDgCol0609A0277 | 6 | 9 | 2 | 8 | 4 | 9 |
| RDgCol0609A0280 | 8 | 3 | 4 | 1 | 6 | 2 |
| RDgCol0609A0281 | 4 | 4 | 3 | 3 | 4 | 4 |
| RDgCol0609A0284 | 6 | 1 | 2 | 1 | 4 | 1 |
| RDgCol0609A0285 | 6 | 4 | 2 | 4 | 4 | 4 |
| RDgCol0609A0287 | 8 | 8 | 8 | 4 | 8 | 6 |
| RDgCol0609A0288 | 12 | 4 | 12 | 2 | 12 | 3 |
| RDgCol0609A0289 | 12 | 4 | 12 | 4 | 12 | 4 |
| RDgCol0609A0291 | 9 | 6 | 9 | 2 | 9 | 4 |
| RDgCol0609A0295 | 12 | 4 | 12 | 4 | 12 | 4 |
| RDgCol0609A0296 | 2 | 12 | 2 | 6 | 2 | 9 |
| RDgCol0609A0297 | 8 | 12 | 8 | 6 | 8 | 9 |
| RDgCol0609A0298 | 9 | 1 | 9 | 1 | 9 | 1 |
| RDgCol0611A0309 | 12 | 6 | 12 | 2 | 12 | 4 |
| RDgCol0611A0311 | 12 | 8 | 6 | 4 | 8 | 6 |
| RDgCol0611A0312 | 9 | 3 | 9 | 1 | 9 | 2 |
| RDgCol0611A0313 | 6 | 6 | 12 | 6 | 9 | 6 |
| RDgCol0611A0314 | 12 | 9 | 12 | 3 | 12 | 6 |
| RDgCol0611A0317 | 8 | 9 | 8 | 3 | 8 | 6 |
| RDgCol0611A0319 | 8 | 6 | 4 | 6 | 6 | 6 |
| RDgCol0611A0324 | 6 | 3 | 6 | 1 | 6 | 2 |
| RDgCol0611A0327 | 12 | 8 | 12 | 4 | 12 | 6 |
| RDgCol0612A0334 | 12 | 6 | 12 | 6 | 12 | 6 |
| RDgCol0612A0335 | 6 | 4 | 6 | 1 | 6 | 3 |
| RDgCol0612A0336 | 9 | 2 | 8 | 2 | 9 | 2 |
| RDgCol0612A0341 | 6 | 4 | 6 | 2 | 6 | 3 |
| RDgCol0612A0343 | 1 | 1 | 1 | 0 | 1 | 1 |
| RDgCol0612A0344 | 3 | 4 | 3 | 3 | 3 | 4 |
| RDgCol0612A0346 | 12 | 12 | 12 | 12 | 12 | 12 |
| RDgCol0612A0348 | 9 | 6 | 6 | 2 | 8 | 4 |
| RDgCol0612A0352 | 6 | 3 | 6 | 1 | 6 | 2 |
| RDgCol0612A0353 | 8 | 2 | 4 | 1 | 6 | 2 |
| RDgCol0612A0356 | 2 | 1 | 1 | 1 | 2 | 1 |
| RDgCol0701A0395 | 4 | 1 | 4 | 1 | 4 | 1 |
| RDgCol0701A0398 | 2 | 1 | 1 | 1 | 2 | 1 |
| RDgCol0701A0402 | 4 | 4 | 4 | 1 | 4 | 3 |
| RDgCol0701A0403 | 9 | 4 | 8 | 3 | 9 | 4 |
| RDgCol0701A0404 | 2 | 6 | 2 | 6 | 2 | 6 |
| RDgCol0701A0405 | 6 | 1 | 6 | 1 | 6 | 1 |
| RDgCol0704A0431 | 4 | 3 | 4 | 2 | 4 | 3 |
| RDgCol0704A0434 | 8 | 2 | 4 | 2 | 6 | 2 |
| RDgCol0704A0437 | 8 | 2 | 4 | 1 | 6 | 2 |
| RDgCol0704A0438 | 12 | 6 | 12 | 6 | 12 | 6 |
| RDgCol0704A0439 | 4 | 1 | 3 | 1 | 4 | 1 |
| RDgCol0704A0441 | 12 | 9 | 12 | 8 | 12 | 9 |
| RDgCol0704A0476 | 8 | 6 | 8 | 6 | 8 | 6 |
| RDgCol0704A0442 | 6 | 8 | 2 | 4 | 4 | 6 |
| RDgCol0704A0475 | 6 | 2 | 6 | 1 | 6 | 2 |
| RDgCol0704A0447 | 4 | 2 | 4 | 1 | 4 | 2 |
| RDgCol0704A0452 | 12 | 4 | 12 | 2 | 12 | 4 |
| RDgCol0704A0455 | 12 | 6 | 12 | 2 | 12 | 4 |
| RDgCol0704A0458 | 4 | 1 | 2 | 1 | 3 | 1 |
| RDgCol0704A0459 | 2 | 4 | 1 | 4 | 2 | 4 |
| RDgCol0704A0460 | 8 | 6 | 8 | 2 | 8 | 4 |
| RDgCol0704A0463 | 8 | 4 | 4 | 4 | 6 | 4 |
| RDgCol0704A0465 | 12 | 4 | 12 | 1 | 12 | 3 |
| RDgCol0704A0466 | 3 | 1 | 3 | 1 | 3 | 1 |
| RDgCol0705A0524 | 9 | 3 | 6 | 2 | 8 | 3 |
| RDgCol0705A0525 | 9 | 4 | 9 | 3 | 9 | 4 |
| RDgCol0705A0528 | 9 | 6 | 3 | 1 | 6 | 4 |
| RDgCol0705A0529 | 3 | 1 | 3 | 1 | 3 | 1 |
| RDgCol0705A0530 | 6 | 2 | 6 | 1 | 6 | 2 |
| RDgCol0705A0531 | 6 | 2 | 2 | 1 | 4 | 2 |
| RDgCol0705A0532 | 8 | 3 | 8 | 2 | 8 | 3 |
| RDgCol0705A0533 | 8 | 2 | 4 | 1 | 6 | 2 |
| RDgCol0705A0535 | 12 | 8 | 12 | 4 | 12 | 6 |
| RDgCol0705A0537 | 9 | 1 | 8 | 1 | 9 | 1 |
| RDgCol0705A0539 | 4 | 2 | 4 | 1 | 4 | 2 |
| RDgCol0705A0540 | 4 | 2 | 4 | 1 | 4 | 2 |
| RDgCol0705A0541 | 6 | 2 | 2 | 1 | 4 | 2 |
| RDgCol0705A0544 | 8 | 1 | 4 | 1 | 6 | 1 |
| RDgCol0706A0575 | 2 | 4 | 1 | 4 | 2 | 4 |
| RDgCol0706A0576 | 1 | 2 | 1 | 1 | 1 | 2 |
| RDgCol0706A0577 | 4 | 1 | 4 | 1 | 4 | 1 |
| RDgCol0706A0579 | 8 | 6 | 8 | 2 | 8 | 4 |
| RDgCol0706A0580 | 12 | 9 | 12 | 6 | 12 | 8 |
| RDgCol0706A0583 | 12 | 9 | 12 | 9 | 12 | 9 |
